# Protein phosphatase 2C-alpha knockdown reduces angiotensin II-mediated skeletal muscle wasting via restoration of mitochondrial recycling and function

**DOI:** 10.1186/2044-5040-4-20

**Published:** 2014-10-30

**Authors:** Alexander Michael Tabony, Tadashi Yoshida, Sergiy Sukhanov, Patrice Delafontaine

**Affiliations:** 1 Tulane University Department of Medicine, Heart and Vascular Institute, New Orleans, LA 70112, USA; 2 Heart and Vascular Institute, Tulane University School of Medicine, 1430 Tulane Ave. SL-48, New Orleans, LA, USA

**Keywords:** Angiotensin II, PP2C, Muscle atrophy, Mitochondria, Autophagy

## Abstract

**Background:**

Circulating angiotensin II (AngII) is elevated in congestive heart failure (CHF), and leads to skeletal muscle wasting, which is strongly associated with poor patient outcomes. We previously found that AngII upregulates protein phosphatase 2C-alpha (PP2Cα) and dephosphorylates AMP-activated protein kinase (AMPK), a critical regulator of cellular metabolism, in skeletal muscle.

**Methods:**

To determine the role of PP2Cα in AngII-induced wasting, gastrocnemius (Gas) muscles of FVB mice were injected with scrambled or PP2Cα siRNA and mice were infused with saline or AngII for 4 days.

**Results:**

Knockdown of PP2Cα reduced AngII wasting, blocked AngII upregulation of PP2Cα, increased p-T172-AMPK, and inhibited AngII-mediated reductions in peroxisome proliferator-activated receptor-γ coactivator-1α (PGC-1α), nuclear respiratory factor 1 (NRF1), mitochondrial transcription factor A (TFAM), in complex IV activity, and in ATP levels. AngII impaired the rate of autophagy as determined by a 2.4-fold increase in p62/SQSTM1 (p62) accumulation. This induction was reduced by PP2Cα knockdown, which also increased beclin-1 expression and microtubule-associated protein 1 light chain 3 (LC3)-II conversion in AngII-infused Gas. AngII reduced activating S555 phosphorylation of UNC-51-like kinase 1 (ULK1), a critical regulator of autophagosome formation, and increased inhibitory S757 ULK1 phosphorylation and these effects were prevented by PP2Cα siRNA.

**Conclusions:**

AngII inhibited AMPK activity and reduced PGC-1α and TFAM expression (thereby inhibiting mitochondrial biogenesis) and impaired ULK1 activation and autophagy (thereby also inhibiting clearance of damaged mitochondria), resulting in mitochondrial dysfunction, decreased ATP, and wasting. Knockdown of PP2Cα normalized AMPK activity, PGC-1α, NRF1, and TFAM levels and blocked AngII inhibition of ULK1, leading to improved mitochondrial biogenesis/recycling/function, energy production, and inhibition of AngII-induced wasting. These results demonstrate novel effects of AngII on cellular metabolism that are likely critical in mediating the muscle wasting that is a hallmark of CHF.

## Background

In conditions such as congestive heart failure and chronic kidney disease, where loss of lean body mass correlates strongly with poor prognosis [1-3], circulating angiotensin II (AngII) is elevated, and is a likely major contributor to skeletal muscle wasting associated with these diseases. Indeed, we have shown that AngII infusion in rodents induces muscle atrophy which is related to multiple mechanisms including activation of the ubiquitin proteasome pathway (UPP) [4,5], inhibition of the insulin/IGF-1/Akt/mammalian target of rapamycin (mTOR) axis [5-13], reduction in appetite [14-16], and activation of apoptosis [10,17]. The effects of AngII include indirect effects via circulating mediators [6,13,18-22] and direct effects to reduce regeneration [16,23].

While it has been well established that AngII causes skeletal muscle atrophy, much less is known about the metabolic and bioenergetic effects of AngII, or how these effects relate to AngII wasting. Protein breakdown in skeletal muscle is predominantly mediated by the UPP and the autophagy-lysosome system, but the specific role of autophagy in AngII skeletal muscle wasting is not known. We originally reported that AngII impaired AMP-activated protein kinase (AMPK) activation in muscle, likely via upregulation of protein phosphatase 2C-alpha (PP2Cα) (AngII did not inhibit kinases upstream to AMPK), leading to mitochondrial dysfunction and ATP depletion, and that direct activation of AMPK with 5-aminoimidazole-4-carboxamide ribonucleoside (AICAR) prevented AngII wasting [14,16]. The aims of this study were to determine the mechanistic link between PP2Cα and AngII wasting and to uncover the mechanisms involved in AngII-induced mitochondrial dysfunction and ATP depletion in skeletal muscle.

## Methods

### *In vivo* muscle siRNA electroporation

Four separate siRNAs targeted to PP2Cα mRNA (SureSilencing™ siRNA set, Qiagen/SA Biosciences) were tested for knockdown efficiency *in vivo*, by injecting gastrocnemius (Gas) muscles of ketamine/xylazine anesthetized FVB mice (male, 8 to 10 weeks old, Charles River) with 2.5 μg [24,25] of each siRNA and control scrambled siRNA in sterile saline at 5 sites using 50 μL Hamilton syringes with 22 gauge needles, followed by immediate electroporation of the muscle using a BTX Harvard Apparatus ECM 830 Electro Square Porator™ with the following settings: 50 V [24,25], 20 ms pulse duration, 0.5 cm between electrodes, 8 unipolar pulses [25,26], and 1 s between pulses. The target sequences of the four PP2Cα siRNAs were as follows: siRNA A - CCAAGATATTTCTGAGACATT, siRNA B - CCAGATACAAATTACCTGTTT, siRNA C - CTTGGTGGATGGGCAGATCTT, and siRNA D - CAAGCTGCAATCATGGAACTT. After determining the most effective siRNA, it was used for time course (1, 3, 5, 7, and 11 days) and dose response experiments (vehicle, 2.5 μg, 5 μg, 7.5 μg, 10 μg, 12.5 μg siRNA) to further optimize target knockdown in Gas *in vivo*.

### Experimental design

In the experimental setting, 5 μg of PP2Cα siRNA and scrambled siRNA were electroporated into contralateral Gas muscles (alternating right and left between animals). Three days later, mice were implanted subcutaneously with osmotic minipumps (Alzet 1007D) continuously infusing either sterile saline (control) or 1,000 ng/kg/min AngII (Phoenix Pharmaceuticals). This dose of AngII yields a 2.8-fold increase in plasma AngII that is within the pathophysiological ranges observed in patients with congestive heart failure (CHF) and chronic kidney disease [12,14,27-30]. Mice were sacrificed after another 4 days, muscles were collected, weighed, and either used fresh or embedded in Allprotect Tissue Reagent (Qiagen) and stored at -80°C until processing. Cryosections were prepared by incubating Gas muscles in 50% optimal cutting temperature (OCT) compound (Tissue-Tek, USA) for 15 min on ice followed by freezing in 100% OCT. Eight micron serial cross sections were prepared from the middle of each muscle and kept at -80°C until processing. The animal protocols were approved by Tulane University Institutional Animal Care and Use Committee.

### Biochemical assays

Frozen tissue was blotted dry of excess Allprotect reagent and pulverized with a mortar and pestle over liquid nitrogen. Aliquots of each pulverized muscle were prepared as previously described [14] for mitochondrial quantification (via DNA isolation followed by real-time PCR using primers specific for mitochondrial DNA and genomic DNA), complex IV activity (Mitochondria Activity Assay Kit, BioChain), ATP quantification (ATP determination kit, Invitrogen), quantitative real-time RT-PCR (using Qiagen/SuperArray optimized primers for PP2Cα, p62/SQSTM1, LC3A, Fbxo32, and Trim63, plus β-actin and HPRT1 housekeeping genes), and SDS-PAGE/western blotting. Primary antibodies utilized were as follows: from Abcam: PPM1A (ab14824), PP2C alpha + PP2C beta (ab27267), PGC-1α (ab54481), Fbxo32 (ab74023), MuRF1 (ab77577), Mitofusin-1, TTC11 (Fis1, ab71498), MARCH5 (ab77585), UCP3 (ab3477), Mitofusin-2 (ab56889), TFAM (ab131607), OPA1 (ab55772), Mff (ab81127), and MitoProfile Total OXPHOS Rodent WB Antibody Cocktail (Abcam/MitoSciences, ab110413), from Cell Signaling Technology: p-T172-AMPKα (2531), AMPKα (2532), p-S473-Akt (4058), p-T308-Akt (5106), Akt (9272), Phospho-Fox01 (Thr24)/Fox03a (Thr32) Antibody (9464), p-S413-Fox03a (8174), Fox01 (2880), Fox03a (2497), caspase-3 (9662), cleaved caspase-3 (9664), TCF11/NRF1 (8052), p-S616-DRP1 (3455), DRP1 (5391), Ubiquitin (3936), Beclin-1 (3495), LC3A/B (12741), LAMP1 (9091), p-S757-Ulk1 (6888), p-S555-Ulk1 (5869), Ulk1 (8054), SQSTM1/p62 (5114), β-Actin, and α-tubulin (2144), from Enzo: Rpt6 (BML-PW9265).

### Immunohistochemistry

Serial skeletal muscle frozen cross sections were stained for LC3 and p62 using rabbit a/LC3 pAb from MBL Co., Ltd (Cat# PM036, Japan) or mouse a/SQSTM1/p62 mAb from Abcam (cat# ab56416, USA), respectively. Sections were fixed with 4% paraformaldehyde (15 min), treated with 0.1% Triton X-100 in PBS, and stained with primary a/b for 1 h at room temperature. Biotin-conjugated anti-mouse (or anti-rabbit) IgG (1:400, Vector Labs, cat# BA2000 and BA1000, respectively) were applied for sections for 45 min followed by incubation with streptavidin-Alexa594 conjugate (1:500) plus DAPI (both from Invitrogen). Antibody specificity was verified routinely by staining of serial sections with ‘normal’ IgG (obtained from an unimmunized animal of the same species as primary antibody, Santa Cruz Biotechnology, Santa Cruz, CA, USA). We did not observe any detectable immunopositivity on ‘normal’ IgG-stained sections (data not shown).

### Statistical analysis

All data are presented as means +/- standard errors (SEM, n indicated in figure legends and within each individual group/bar). GraphPad software (Version 6.03 for Windows, GraphPad Software, San Diego, CA, USA) was used to perform the statistical analysis. Differences between groups were determined by two-way ANOVA followed by Holm-Sidak’s multiple comparisons test (for experiments involving both AngII infusion and siRNA intervention, as well as for time courses), or by one-way ANOVA followed by Tukey post-tests (determination of optimal siRNA target sequence and dose responses with a single siRNA) as appropriate. A value of *P* <0.05 was considered statistically significant.

## Results

### Knockdown of PP2Cα *in vivo*

**Figure 1:**
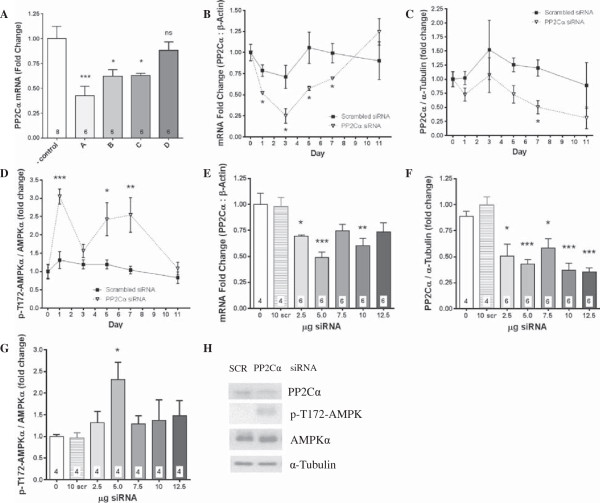
***In vivo *knockdown of PP2Cα.** The indicated siRNAs were injected at five sites in Gas muscles which were then electroporated to induce uptake of the siRNA. The most effective sequence, time point, and dose of siRNA were chosen for subsequent AngII-infusion experiments. **(A)** Real-time PCR showing PP2Cα mRNA knockdown in Gas using 2.5 μg of four different PP2Cα siRNA target sequences relative to negative control siRNA at day 4 post electroporation. PP2Cα siRNA ‘A’ was used in all time course and dose response experiments. **(B)** Time-course of PP2Cα mRNA knockdown in Gas with 2.5 μg of scrambled and PP2Cα siRNA. **(C)** Time-course of PP2Cα protein knockdown in Gas with 2.5 μg of scrambled and PP2Cα siRNA. **(D)** Time-course of AMPK phosphorylation in Gas with 2.5 μg of scrambled and PP2Cα siRNA. **(E)** Dose response of PP2Cα mRNA knockdown in Gas at day 7 post electroporation. **(F)** Dose response of PP2Cα protein knockdown in Gas at day 7 post electroporation. **(G)** Dose response of AMPK phosphorylation in Gas at day 7 post electroporation. **(H)** Representative western blots showing PP2Cα protein knockdown and AMPK activation with 5 μg of PP2Cα siRNA at day 7. n =4-8 per group, Mean ± SEM, *P <0.05, **P <0.01, ***P <0.001.

Four days after electroporation, PP2Cα siRNAs B and C both significantly reduced expression of PP2Cα mRNA in Gas by approximately 37% (*P* <0.05), while siRNA A proved to be the most effective with a 57% reduction (*P* <0.001) in mRNA compared to control, and siRNA D failed to reduce expression of PP2Cα (Figure 1A). A total of 2.5 μg of PP2Cα siRNA A significantly reduced gene expression from 1 to 7 days post electroporation, with a peak suppression from baseline of 75% measured at day 3, and returning to normal by day 11 (Figure 1B). There was a non-statistically significant trend for transient suppression of PP2Cα mRNA expression with scrambled siRNA/electroporation, which returned to normal between days 3 and 5 (Figure 1B). Similarly, PP2Cα protein tended to be reduced by PP2Cα siRNA A throughout the time course, with a significant reduction of 58% at day 7 (Figure 1C), while AMPK phosphorylation was significantly increased at days 1, 5, and 7 (Figure 1D). The dose response performed at day 7 indicated that 5 μg of siRNA was the optimal dose, with a 51% reduction in expression compared to 31% with 2.5 μg, while scrambled siRNA did not reduce expression at all (Figure 1E). PP2Cα protein expression was significantly reduced at all doses of PP2Cα siRNA (Figure 1F), and there was a concomitant 2.3-fold increase in AMPK phosphorylation with 5 μg of PP2Cα siRNA (Figure 1G).

### PP2Cα knockdown prevented AngII skeletal muscle wasting

**Figure 2:**
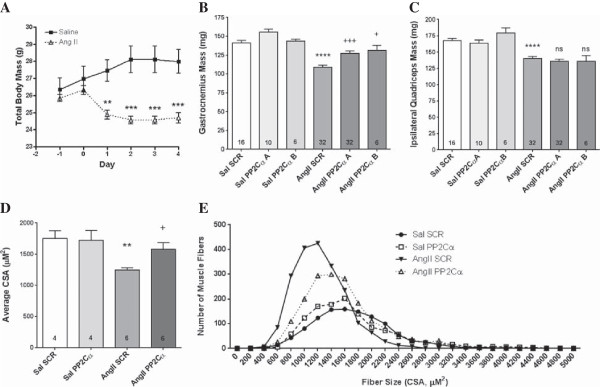
**PP2Cα knockdown prevented AngII wasting.** A total of 5 μg of the indicated siRNA sequences were injected at five sites in Gas muscles which were then electroporated to induce uptake of the siRNA. Three days later, osmotic minipumps were implanted subcutaneously and continually infused either saline or 1,000 ng/kg/min AngII for another 4 days. **(A)** Total body mass showing whole-body AngII wasting over 4 days of continuous infusion. **(B)** Gastrocnemius wet weight showing AngII muscle wasting and rescue with PP2Cα knockdown at 4 days. Two PP2Cα siRNA target sequences (A and B, Figure 1A) were tested to verify the specificity of the rescue effect. **(C)** Quadriceps wet weight showing AngII muscle wasting after 4 days. Quadriceps were not treated with siRNA, so quadriceps muscles ipsilateral to Gas receiving PP2Cα siRNA showed no rescue effect. **(D)** Average cross sectional area of Gas muscle fibers infused with either saline or AngII and treated with either scrambled or PP2Cα siRNA ‘A’. **(E)** CSA frequency distribution curves of Gas muscle fibers. n =6-32 per group, Mean ± SEM, **P <0.01, ***P <0.001, ****P <0.0001 (Saline scrambled vs. AngII scrambled), +*P* <0.05, +++*P* <0.001 (AngII scrambled vs. AngII PP2Cα siRNA).

AngII-infused mice lost 12.5% of total body mass (*P* <0.001) and 26% of Gas mass (*P* <0.001) compared to saline-infused controls at day 4 (Figure 2A and B). Two unique PP2Cα siRNAs significantly rescued the AngII-induced reduction in Gas mass by approximately 37% (*P* <0.001 and *P* <0.05 respectively, Figure 2B). As an additional control, we measured weights of ipsilateral quadriceps muscles, which were not treated with siRNA. As expected, there was no rescue in the quadriceps of the legs receiving either PP2Cα siRNA in Gas (Figure 2C). AngII also reduced average cross sectional area of individual Gas muscle fibers (Figure 2D) and induced a frequency shift towards smaller fibers (Figure 2E), and PP2Cα siRNA prevented the AngII-induced reductions in fiber size (Figure 2D and E).

### PP2Cα knockdown restored AMPK activation and PGC-1α expression to activate mitochondrial biogenesis

**Figure 3:**
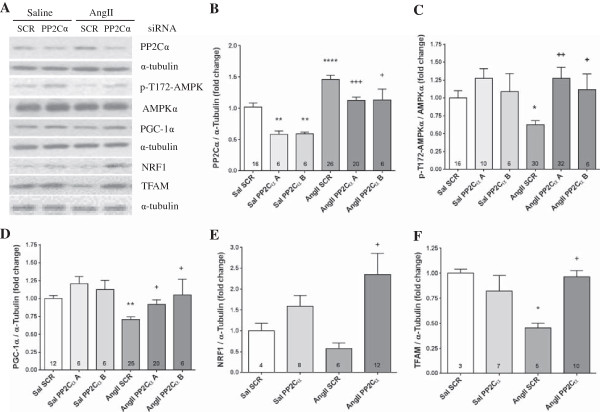
**PP2Cα knockdown restored AMPK activation and signaling related to mitochondrial biogenesis.** Specificity was ensured by performing experiments with two siRNA target sequences, as indicated. **(A)** Representative western blots showing effects of AngII and PP2Cα siRNA ‘A’ on the AMPK-PGC-1α-TFAM signaling axis. **(B)** PP2Cα protein expression was increased with AngII, which was prevented with PP2Cα siRNA. **(C)** Activating AMPK phosphorylation was reduced with AngII and increased with PP2Cα knockdown. **(D)** PGC-1α protein expression was reduced with AngII, which was prevented with PP2Cα siRNA. **(E)** PP2Cα siRNA increased expression of NRF1 transcription factor. **(F)** AngII reduced expression of the mitochondrial transcription factor TFAM, and this reduction was prevented by PP2Cα siRNA. n =4-32 per group, Mean ± SEM, *P <0.05, **P <0.01, ****P <0.0001 (Saline scrambled vs. AngII scrambled), +*P* <0.05, +++*P* <0.001 (AngII scrambled vs. AngII PP2Cα siRNA).

Infusion of AngII caused a 46% increase in PP2Cα protein expression (*P* <0.0001), consistent with our previous findings [14], and two separate PP2Cα siRNAs significantly reduced basal PP2Cα expression and blocked the AngII-mediated increase (down to a 13% increase, *P* <0.001 and *P* <0.05 respectively, Figure 3B). Consistent with increased PP2Cα expression, AngII reduced activating T172 AMPK phosphorylation by 37% (*P* <0.05, Figure 3C), and PP2Cα knockdown with two unique siRNAs increased AMPK phosphorylation by 2-fold and 1.8-fold respectively compared to scrambled siRNA (Figure 3C). Active AMPK increases expression of PGC-1α, which is a critical mediator of mitochondrial biogenesis, increasing the expression of transcription factors NRF1 and TFAM, which coordinate expression of mitochondrial genes and are critical for transcription of mitochondrial DNA (mtDNA) [31-33]. PGC-1α expression was reduced by 29% with infusion of AngII (*P* <0.001, Figure 3D), with a near complete rescue by PP2Cα knockdown using two separate siRNAs (*P* <0.05, Figure 3D). Similarly, AngII also tended to reduce expression of NRF1 (Figure 3E), and significantly reduced TFAM (Figure 3F), while PP2Cα knockdown significantly increased expression of both NRF1 (Figure 3E) and TFAM (Figure 3F). Together, these results indicate that knockdown of PP2Cα prevented the AngII-mediated suppression of mitochondrial biogenesis.

### PP2Cα siRNA-mediated rescue of AngII wasting is independent of the Akt-FOXO signaling axis

**Figure 4:**
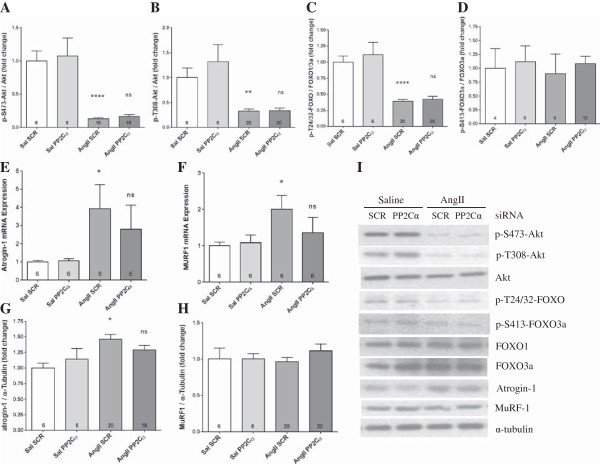
**PP2Cα knockdown did not restore AngII-mediated reductions in Akt or Fox0 phosphorylation or increased E3 ubiquitin ligase expression.** All data were generated from experiments utilizing PP2Cα siRNA ‘A’. **(A, B)** Activating Akt phosphorylation was reduced with AngII, but not rescued by PP2Cα knockdown. **(C)** Akt-mediated inhibitory Fox0 phosphorylation was reduced with AngII, but not rescued by PP2Cα knockdown. **(D)** AMPK-mediated phosphorylation of FOXO3a was not altered by AngII or PP2Cα knockdown. **(E)** Expression of atrogin-1 mRNA was upregulated by AngII, but not rescued by PP2Cα knockdown. **(F)** Expression of MURF1 mRNA was upregulated by AngII, but not rescued by PP2Cα knockdown. **(G)** Expression of atrogin-1 protein was upregulated by AngII, but not rescued by PP2Cα knockdown. **(H)** No change in MURF1 protein expression was detected with AngII or PP2Cα siRNA. **(I)** Representative western blots showing effects of AngII and PP2Cα siRNA ‘A’ on the Akt-FOXO-E3 ligase signaling axis. n =6-20 per group, Mean ± SEM, *P <0.05, ***P <0.001 (Saline scrambled vs. AngII scrambled), +*P* <0.05 (AngII scrambled vs. AngII PP2Cα siRNA).

Although AngII greatly reduced activating S473 and T308 Akt phosphorylation (by 87% and 67%, respectively, *P* <0.001, Figure 4A and B), as well as downstream Akt-mediated FoxO1/FoxO3a phosphorylation (by 61%, *P* <0.001, Figure 4C), PP2Cα knockdown did not reverse those reductions, and had no effect on basal Akt and FOXO phosphorylation. Consistent with Akt and FOXO phosphorylation status, AngII also significantly increased gene expression of the E3 ubiquitin ligases atrogin-1 (Figure 4E), and MuRF1 (Figure 4F), while also increasing expression of atrogin-1 protein (Figure 4G), but not MURF1 protein (Figure 4H). PP2Cα knockdown did not significantly reduce AngII-mediated induction of E3 ligase expression, and had no effect on basal expression of these two E3 ligases (Figure 4E-H). Further, phosphorylation at S413-FOXO, which is mediated by AMPK was not altered with AngII or PP2Cα knockdown (Figure 4D). Since knockdown of PP2Cα did not prevent Akt inhibition, FOXO activation, and increased expression of atrogin-1 or MURF1 by AngII, other pathways must be involved in the ability of PP2Cα knockdown to reduce AngII-induced wasting.

### PP2Cα knockdown increased mitochondrial content and ameliorated AngII-induced mitochondrial dysfunction

**Figure 5:**
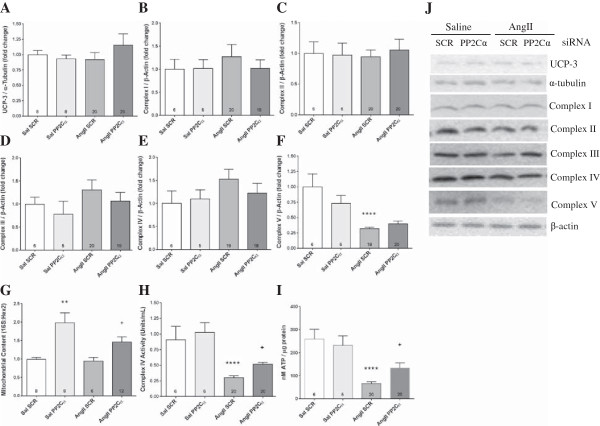
**PP2Cα knockdown increased mitochondrial content and partially restored AngII-induced mitochondrial dysfunction.** All data were generated from experiments utilizing PP2Cα siRNA ‘A’. **(A)** UCP-3 expression, **(B)** NADH dehydrogenase (complex I) expression, **(C)** succinate dehydrogenase (complex II) expression, **(D)** cytochrome bc_1_ complex (complex III) expression, and **(E)** cytochrome C oxidase (complex IV) expression were not altered by AngII or PP2Cα siRNA. **(F)** AngII reduced ATP synthase (complex V) expression, which was not restored with PP2Cα siRNA. **(G)** Relative mitochondrial copy number was unaltered by AngII, but PP2Cα siRNA increased mitochondrial load. **(H)** Complex IV (Cytochrome C Oxidase) activity was reduced with AngII and partially restored with PP2Cα knockdown. **(I)** ATP was reduced with AngII and partially restored with PP2Cα knockdown. **(J)** Representative western blots showing expression of ETC., complexes I-V, and uncoupling protein-3. n =6-20, Mean ± SEM, ***P <0.001 (Saline scrambled vs. AngII scrambled), +*P* <0.05 (AngII scrambled vs. AngII PP2Cα siRNA).

We have previously shown that AngII reduced skeletal muscle ATP levels, which could be prevented by activating AMPK with AICAR [14]. Therefore, we analyzed parameters of mitochondrial content and function with PP2Cα knockdown. There was no observed change in the expression of uncoupling protein-3 (UCP3), or of electron transport chain complexes I-IV with AngII or PP2Cα knockdown (Figure 5A-E), but AngII reduced ATP-synthase (complex V) expression by 68% (*P* <0.0001, Figure 5G), and PP2Cα siRNA did not restore complex V expression to normal (Figure 5F). Although AngII did not alter mitochondrial copy number compared to control, knockdown of PP2Cα increased basal mitochondrial content by 98% and by 46% in AngII infused Gas compared to scrambled siRNA, consistent with the trends observed in expression of PGC-1α (*P* <0.05, Figure 5G).

Expression of cytochrome C oxidase (complex IV) was not reduced (if anything there was a trend for increased expression with AngII, Figure 5E), but complex IV activity was reduced by 70% with AngII (*P* <0.0001), and PP2Cα knockdown partially blocked the effect of AngII such that complex IV activity was reduced by only 48% (*P* <0.05, Figure 5H). Quantification of ATP in Gas revealed a parallel trend, with a 74.5% reduction with AngII (*P* <0.0001), which was partially inhibited to a 49% reduction by PP2Cα knockdown (*P* <0.05, Figure 5I). PP2Cα knockdown did not alter complex IV activity or ATP content in the absence of AngII.

**Figure 6:**
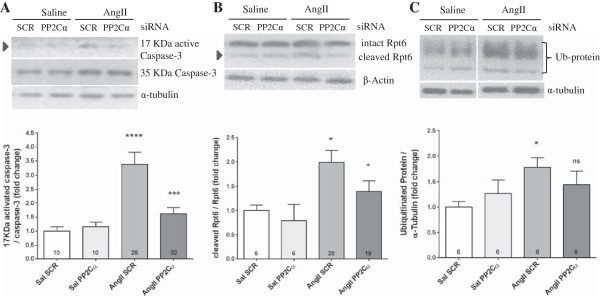
**PP2Cα knockdown prevented caspase-3 activation and Rpt6 cleavage.** All data were generated from experiments utilizing PP2Cα siRNA ‘A’. **(A)** 17 KDa activated caspase-3 was increased by AngII, and this increase was prevented by PP2Cα knockdown. **(B)** The proteasome 19S cap ATP-ase Rpt6 (a target of caspase-3) cleavage was increased with AngII, and this cleavage was blunted by PP2Cα knockdown. **(C)** Ubiquitin-conjugated protein was increased with AngII, but not significantly reduced by PP2Cα siRNA. n =6-32 per group, Mean ± SEM, *P <0.05, ****P <0.0001 (Saline scrambled vs. AngII scrambled), +*P* <0.05, +++*P* <0.001 (AngII scrambled vs. AngII PP2Cα siRNA).

Prolonged mitochondrial dysfunction and bioenergetic stress can lead to initiation of apoptosis, which is characterized by caspase-3 release and activation [34,35], and activation of caspase-3 is known to be required for AngII wasting [4,17]. Expression of total caspase-3 did not change with AngII or PP2Cα knockdown, but 17KDa cleaved activated caspase-3 was increased 3.4-fold with AngII (*P* <0.0001) and PP2Cα siRNA reduced that increase to only 1.6-fold, while not changing basal caspase activation (*P* <0.001, Figure 6A). Because caspase-3 activation has been linked to proteasome activation via cleavage of the 19S regulatory cap ATP-ase Rpt6 [17], we also assessed Rpt6 cleavage with AngII and PP2Cα knockdown. AngII increased Rpt6 cleavage by 2-fold without altering total Rpt6 expression (*P* <0.05), and that increased cleavage was blunted by PP2Cα siRNA (*P* <0.05, Figure 6B). PP2Cα knockdown in saline controls had no effect on Rpt6 cleavage. Ubiquitin-conjugated proteins were also increased with AngII, indicating an increase in substrates for the proteasome, while this increase tended to be reduced with PP2Cα knockdown but did not approach significance (Figure 6C).

Collectively, these data indicate that AngII caused profound mitochondrial dysfunction without significantly altering total mitochondrial load, while also implicating activation of the proteasome. Mitochondrial copy number is coordinated simultaneously by mitochondrial biogenesis and mitochondrial turnover via mitophagy. As such, the fact that AngII did not alter mitochondrial content compared to control while significantly inhibiting pathways critical to mitochondrial biogenesis implicates an effect of AngII on autophagy/mitophagy. AngII-induced mitochondrial dysfunction was associated with activation of apoptotic signaling as evidenced by caspase-3 activation. Further, PP2Cα knockdown partially prevented AngII-induced mitochondrial dysfunction, caspase-3 activation, and Rpt6 cleavage.

### PP2Cα siRNA prevented AngII-mediated inhibition of autophagy

Healthy mitochondrial function is preserved by balancing mitochondrial biogenesis and recycling via mitophagy. Since AngII reduced PGC-1α without reducing total mitochondrial load in Gas, this indicated a potential impairment in mitophagy. To evaluate the role of autophagy in AngII-mediated mitochondrial dysfunction and wasting, we quantified several autophagy markers, including p62 accumulation, beclin-1 expression, and LC3-II conversion.

**Figure 7:**
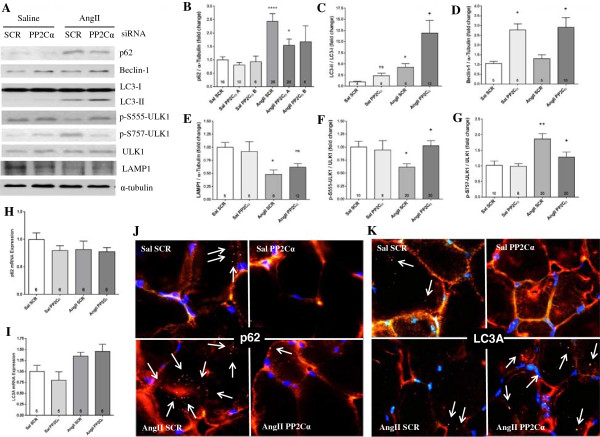
**AngII inhibited autophagy while PP2Cα knockdown activated it via ULK1. (A)** Representative western blots showing effects of AngII and PP2Cα siRNA on markers of autophagy. **(B)** AngII increased p62 accumulation (indicative of an inhibition of autophagy) and knockdown of PP2Cα prevented p62 accumulation. Specificity of the rescue was verified via utilization of two PP2Cα siRNAs, as indicated. All other data were generated from experiments utilizing PP2Cα siRNA ‘A’. **(C)** LC3-II conversion was increased with AngII, but increased further by PP2Cα knockdown. **(D)** PP2Cα siRNA increased expression of the autophagy marker Beclin-1. **(E)** AngII reduced expression of the lysosome marker LAMP1, but this reduction was not restored by PP2Cα siRNA. **(F)** AngII decreased activating ULK1 phosphorylation, which was prevented by PP2Cα siRNA. **(G)** AngII increased inhibitory ULK1 phosphorylation, which was prevented by PP2Cα siRNA. **(H, I)** There was no transcriptional regulation of p62 **(H)**, or LC3A **(I)** with either AngII or PP2Cα siRNA. **(J)** Representative immunohistochemical images showing the AngII-mediated increase in p62-positive punctae, and the prevention of that increase via knockdown of PP2Cα, showing inhibition of autophagy with AngII and restoration of autophagic flux with PP2Cα knockdown. **(K)** Representative immunohistochemical images showing PP2Cα siRNA increased autophagosome number as determined by LC3-positive punctae. n =4-20, Mean ± SEM, *P <0.05, **P <0.01, ****P <0.0001 (Saline scrambled vs. AngII scrambled), +*P* <0.05 (AngII scrambled vs. AngII PP2Cα siRNA).

Expression of p62 protein was increased by 2.4-fold with AngII (*P* <0.0001), and PP2Cα siRNA blunted that increase to 1.5-fold, while not altering basal p62 (*P* <0.05, Figure 7A and B), and neither AngII nor PP2Cα siRNA had any effect on transcription of p62 (Figure 7H), indicating an inhibition of autophagic flux with AngII and prevention of that inhibition with knockdown of PP2Cα. This is corroborated by the 2.8-fold increase in LC3-II conversion with PP2Cα knockdown over AngII alone, indicative of increased mature autophagosome formation with PP2Cα knockdown (*P* <0.05, Figure 7A and C). LC3A mRNA expression (Figure 7I) was not significantly altered by either AngII or PP2Cα siRNA, indicating that the increased LC3 conversion observed with PP2Cα knockdown (Figure 7C) was not likely mediated by increased transcription of LC3.

Western blot data for p62 were verified by immunofluorescence on Gas cross sections showing an increase in p62-positive punctae with AngII and a reduction compared to AngII alone with knockdown of PP2Cα (Figure 7J). Further, PP2Cα siRNA increased the number LC3A-positive punctae compared to AngII alone (Figure 7K), indicating a greater number of autophagosomes with knockdown of PP2Cα in AngII-infused Gas. PP2Cα siRNA had no obvious effect on the number of p62 or LC3A-positive punctae in saline infused controls (Figure 7J and K). PP2Cα siRNA also increased expression of beclin-1 by 2.8-fold (*P* <0.05, Figure 7A and D), further supporting increased autophagy with PP2Cα knockdown, while AngII reduced expression of the lysosome marker lysosomal-associated membrane protein 1 (LAMP1) indicating that there may be a reduction in the number of lysosomes with AngII, but this reduction was not prevented by PP2Cα siRNA (Figure 7A and E).

Given the observed changes in autophagy markers, ULK1 phosphorylation status was ascertained to obtain potential insights into mechanisms. ULK1 is an upstream mediator of autophagosome formation that is critical for autophagy, and its activity is known to be regulated by AMPK [36-39]. AngII reduced activating S555 phosphorylation of ULK1 by 39% (*P* <0.05), and this effect was completely reversed by PP2Cα siRNA (*P* <0.05, Figure 7F). Inhibitory S757 phosphorylation of ULK1 was increased by 86% with AngII (*P* <0.01), and this increase was also almost completely reversed by knockdown of PP2Cα (*P* <0.05, Figure 7G).

### AngII reduced expression of markers of mitochondrial fusion and fission via AMPK and MARCH5-indepentdent mechanisms

**Figure 8:**
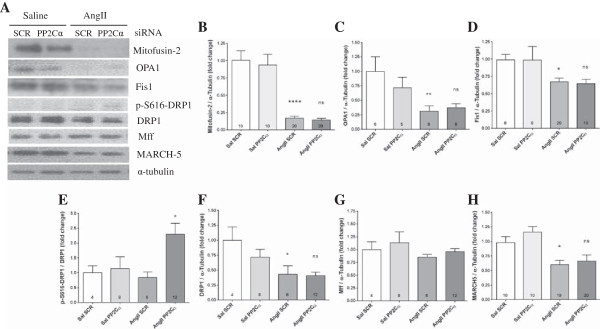
**AngII inhibited expression of mitochondrial fission and fusion proteins via a MARCH5-independent mechanism.** All data were generated from experiments utilizing PP2Cα siRNA ‘A’. **(A)** Representative western blots showing effects of AngII and PP2Cα siRNA on markers of mitochondrial fusion and fission. **(B)** AngII reduced Mitofusin-2 expression, which was not prevented by PP2Cα knockdown. **(C)** AngII reduced OPA1 expression, which was not prevented by PP2Cα knockdown. **(D)** AngII reduced Fis1 expression, which was not prevented by PP2Cα knockdown. **(E)** S616 phosphorylation of DRP1 was increased with PP2Cα siRNA, which facilitates mitochondrial fission. **(F)** DRP1 expression was reduced by AngII, and this was not rescued by PP2Cα knockdown. **(G)** Mff expression was not altered by AngII or PP2Cα siRNA. **(H)** MARCH5 expression was not induced with AngII and was unchanged by PP2Cα siRNA. n =4-20, Mean ± SEM, *P <0.05, **P <0.01, ***P <0.001 (Saline scrambled vs. AngII scrambled), +*P* <0.05 (AngII scrambled vs. AngII PP2Cα siRNA).

Mitofusin-2 and Optic Atrophy 1 (OPA1) facilitate fusion of the outer and inner mitochondrial membranes, respectively. Mitochondrial fission is mediated by mitochondrial fission 1 protein (Fis1), mitochondrial fission factor (Mff), and dynamin-1-like protein (DRP1). Interestingly, Mitofusin-2, OPA1, Fis1, and DRP1 expression were all reduced by Ang II, indicating that Ang II inhibits both mitochondrial fusion and fission. AngII markedly reduced expression of mitofusin-2 (83% decrease, *P* <0.0001), although PP2Cα siRNA did not blunt this decrease (Figure 8A and B). Similarly, AngII also reduced expression of OPA1 by 68% (*P* <0.01) and knockdown of PP2Cα did not prevent this reduction (Figure 8A and C). AngII reduced expression of Fis1 by 33% (*P* <0.05, Figure 8A and D) and of DRP1 by 57% (*P* <0.05, Figure 8A and F), while PP2Cα knockdown did not prevent these reductions (Figure 8A,D,F). PP2Cα knockdown in saline-infused controls had no effect on expression of mitofusin-2, OPA1, DRP1, or Fis1 (Figure 8A-D,F). Neither AngII nor PP2Cα knockdown had any effect on expression of Mff (Figure 8G). DRP1 facilitates mitochondrial fission both via changes in expression and phosphorylation status [40], and while PP2Cα siRNA had no effect on total DRP1 expression, it significantly increased activating DRP1 phosphorylation in AngII-infused Gas (Figure 8A and E). Expression of membrane-associated RING-CH5 (MARCH5), an E3 ubiquitin ligase known to regulate both mitofusin-2 and Fis1 [41,42], was significantly reduced by AngII (Figure 8A and H), indicating that some other mechanism must be responsible for the reductions in mitofusin-2 and Fis1 with AngII. MARCH5 expression was not significantly altered by PP2Cα knockdown in saline or AngII-infused groups (Figure 8A and H).

## Discussion

Our results indicate that PP2Cα is a critical mediator of AngII wasting. Knockdown of PP2Cα via electroporation of siRNA into Gas partially prevented AngII wasting, which is consistent with activation of AMPK being protective against wasting caused by AngII, as we have previously demonstrated [14]. Although there are technical limitations of siRNA electroporation into the skeletal muscle (that is, electroporation efficiency and siRNA effectiveness on PP2Ca mRNA), our PP2Ca knockdown significantly prevented Ang II wasting *in vivo*. Still, some components of AngII-wasting are PP2Cα/AMPK-independent, or at least indirectly related, which may explain the partial rescue. We also demonstrate that the metabolic effects of AngII are critical, as PP2Cα knockdown did not significantly alter the effects of AngII on Akt, FOXO, or expression of E3 ubiquitin ligases atrogin-1 or MURF1. This indicates that something other than the Akt-FoxO-E3 axis is predominately responsible for the rescue effects of PP2Cα knockdown with respect to mitochondrial function, ATP, and muscle wasting. Finally, we have uncovered novel mechanisms linking elevated AngII to mitochondrial dysfunction in skeletal muscle. These mechanisms are both AMPK-dependent (reduced mitochondrial biogenesis via reduction in PGC-1α/TFAM, and inhibition of mitophagy via inhibition of ULK1), and AMPK-independent (reductions in expression of important regulators of mitochondrial network dynamics).

Certainly, the UPP is very important for AngII wasting, and we have previously shown that AngII-mediated effects on Akt/FOXO phosphorylation and upregulation of atrogin-1 and MURF1 mRNA was prevented by direct AMPK activation with AICAR [14]. It is important to point out that the experimental model in the present study may have some differences from our previously published work in that injection and electroporation of Gas muscles represents significant injury to the muscle, and may have induced the muscle’s normal regenerative responses independent of AngII and/or PP2Cα knockdown. Even so, while knockdown of PP2Cα did not significantly rescue from AngII-induced upregulations of atrogin-1, MURF1, and ubiquitin-conjugated proteins, there was a trend for a reduction in each of these endpoints, although these trends remain independent of the Akt-FOXO signaling axis. Further studies will be required to more closely characterize the role of AngII-mediated AMPK inhibition on the UPP, but we have clearly shown that many of the effects of AngII on mitochondrial dysfunction are dependent on AMPK.

As AMPK is activated, PGC-1α expression increases, in turn activating a program of mitochondrial biogenesis [31,32]. As such, AngII’s effect to suppress AMPK phosphorylation and expression of PGC-1α/NRF1/TFAM in skeletal muscle impairs generation of new mitochondria. The deleterious effects of AngII on mitochondrial biogenesis are reflected by AngII’s marked suppression of mitochondrial ATP synthase (complex V) expression, complex IV activity, and ATP generation in Gas. Conversely, the ability of PP2Cα knockdown to increase PGC-1α, NRF1, and TFAM expression acts in opposition to AngII by facilitating mitochondrial biogenesis and PP2Cα siRNA partially restored mitochondrial activity and ATP toward basal levels, while significantly increasing mitochondrial copy number.

Healthy mitochondrial function is normally maintained both by generation of new mitochondria via PGC-1α-NRF1-TFAM-mediated biogenesis, and by sequestration of damaged or defective mitochondria to autophagosomes and their subsequent degradation following fusion to lysosomes in a process known as mitophagy [43,44]. The specificity of mitophagy is mediated via mitochondrial fission and fusion, as well as by several mitophagy proteins, including p62, which binds ubiquitinated mitochondrial proteins and LC3 on the autophagosome to facilitate selective degradation of damaged mitochondria [44,45]. Because p62 is normally degraded along with the rest of the components of the autophagosome, its accumulation in the absence of transcriptional regulation indicates an inhibition of autophagy [44].

AngII significantly increased p62 protein without altering mRNA levels, indicating impairment of autophagy. While p62 clearance/accumulation is a reasonable index of autophagic flux in the absence of transcriptional regulation, it will be informative to measure flux directly in future studies, for instance by tracking LC3 turnover alongside p62 accumulation [46]. The partial restoration of p62 to basal levels, along with increased expression of Beclin-1 and conversion of LC3-II with PP2Cα knockdown, indicates that AngII decreased autophagy via inhibition of AMPK. LC3-II conversion is an important early step in autophagosome formation and a higher ratio of LC3-II to LC3-I indicates a greater number of autophagosomes and usually indicates an induction of autophagy, although increased LC3 expression and conversion can also indicate an inhibition of downstream autophagic processes [47]. Because AngII appeared to reduce the expression of the lysosome marker LAMP1, the increased LC3-II conversion with AngII compared to baseline may implicate such a downstream inhibition in addition to the upstream effects on ULK1. Given that AngII increased p62 accumulation while leaving beclin-1 expression unchanged, the AngII-mediated increase in LC3-II conversion over control likely does not indicate an activation of autophagy over basal rates. Indeed, while it did not reach statistical significance, there appears to be a modest trend for increased LC3A gene expression with AngII, which may also explain the increase in LC3-II conversion over basal conditions with AngII. Knockdown of PP2Cα in the presence of AngII further increased LC3-II conversion, but reduced p62 and also increased beclin-1, indicating that AngII-mediated inhibition of AMPK inhibited autophagy.

Failure of the cell to clear damaged mitochondrial proteins results in the accumulation of dysfunctional mitochondria and a reduced ability to meet cellular energetic demands. This deficit is accentuated in our model (where AMPK activation is impaired by AngII) because AMPK activation is the normal physiologic response to decreased ATP availability. Although AMPK activation can be associated with mitochondrial dependent muscle wasting, and inhibition of AMPK has been shown to restore muscle size in myofibers with altered mitochondria [45], the opposite appears to be the case with regards to wasting caused by AngII [14].

In the current study, p62 accumulation with AngII indicates impairment in the rate of autophagy. This is corroborated by the accumulation of dysfunctional complex IV in AngII-infused muscle, as demonstrated by the marked reduction in complex IV activity, even while total complex IV expression tended to increase. The accumulation of dysfunctional mitochondria is reflected in the reduction of ATP in response to AngII while mitochondrial load remained unchanged. Activation of AMPK signaling via knockdown of PP2Cα acted in opposition to AngII by facilitating autophagy as evidenced by reduced p62 accumulation, and improved mitochondrial function as indicated by increased complex IV activity, and increased ATP. Further, the degree to which PP2Cα siRNA activated mitochondrial biogenesis outweighed the degree to which mitophagy was activated since total mitochondrial content was increased by knockdown of PP2Cα. As such, the net effect of PP2Cα knockdown during infusion of AngII is a greater number of total mitochondria and a simultaneously higher proportion of fully functional, more efficient mitochondria, leading to increased energy production and protection from AngII wasting.

ULK1 is a critical mediator of autophagy [36-39], acting at the most upstream stages of nucleation and initial autophagosome formation. The ULK1 complex is differentially activated and inhibited by AMPK and mTORC1, respectively [36-39,44,47]. AngII reduced activating ULK1 phosphorylation, while simultaneously inducing inhibitory ULK1 phosphorylation in Gas, effects which are likely mediated, at least in part, by AngII’s inhibitory effects on AMPK in muscle, and which serve to inhibit autophagy in muscle. Activation of AMPK via knockdown of its phosphatase PP2Cα alleviated the inhibitory effect of AngII on AMPK and restored ULK1 phosphorylation status to basal levels, in turn restoring autophagic flux to normal levels. The ability of PP2Cα knockdown to oppose the inhibitory effects of AngII on ULK1 activation is consistent with a mechanism whereby AngII impairs autophagy and AMPK acts in opposition to activate it in skeletal muscle.

Parallel to its inhibitory effects on the PGC-1α-NRF1-TFAM axis (mitochondrial biogenesis), ULK1 (autophagosome formation), and p62 clearance (autophagic flux), AngII also significantly reduced expression of several markers of mitochondrial fusion (mitofusin-2, OPA1) and fission (Fis1, DRP1), suggesting that AngII also acts to inhibit both mitochondrial fusion and mitochondrial fission. Mitofusin-2 is located on the outer mitochondrial membrane, while OPA1 is localized to the inner mitochondrial membrane and both are critical for the initiation of mitochondrial fusion [41,44,48]. Fusion of mitochondria into complex networks helps to increase overall mitochondrial efficiency by compensating for damaged or dysfunctional oxidative phosphorylation machinery and tends to be induced during times of high energetic demand [44]. Therefore, inhibition of mitochondrial fusion by AngII would prevent the muscle from compensating for the deleterious effects of AngII on energy balance, and could play a role in ATP depletion with AngII. Fis1 and Mff are localized to the outer mitochondrial membrane, while DRP1 associates with these proteins to form a complex which facilitates fission of the mitochondrial network [40-42,48]. Both DRP1 expression and activity are important for mitochondrial fission, and its activity is modulated via phosphorylation by several upstream kinases including Cdk1/cyclin B, and protein kinase A [49]. Mitochondrial fission is important because it allows the cell to group damaged mitochondrial components together so that they can be selectively recycled via mitophagy (thereby leaving functioning mitochondria intact). The ability of AngII to reduce Fis1 and DRP1 expression may further facilitate AngII-impairment of selective mitophagy and could also help explain the build-up of damaged mitochondrial proteins and energy depletion in response to AngII, while increased activating DRP1 phosphorylation with PP2Cα siRNA may act in opposition to facilitate fission and selective mitophagy.

MARCH5 is an E3 ligase that regulates the expression of both mitofusin-2 and Fis1 via UPP-mediated degradation of these proteins [41]. Its expression is not significantly increased with AngII. In fact, MARCH5 expression was significantly reduced by AngII, making it unlikely that MARCH5 mediates the AngII-induced reductions in mitofusin-2 and Fis1. Further, knockdown of PP2Cα reversed the effects of AngII on PGC-1α and autophagy, but did not reverse the effects of AngII on mitofusin-2, OPA1, DRP1, or Fis1 expression, suggesting that AMPK is not directly involved in mediating the expression of these proteins, and that the ability of PP2Cα knockdown to improve mitochondrial recycling is primarily through restoration of ULK1 activity and autophagosome formation rather than via alterations in mitochondrial fission/fusion. Still, the overall relationship between AngII, AMPK, and the dynamic remodeling of the mitochondrial network will require further studies, since PGC-1α [50,51] and FOXO3a [44] have been shown to play a role in mitochondrial fusion and fission, respectively, and both are known to be regulated by AMPK.

## Conclusions

**Figure 9:**
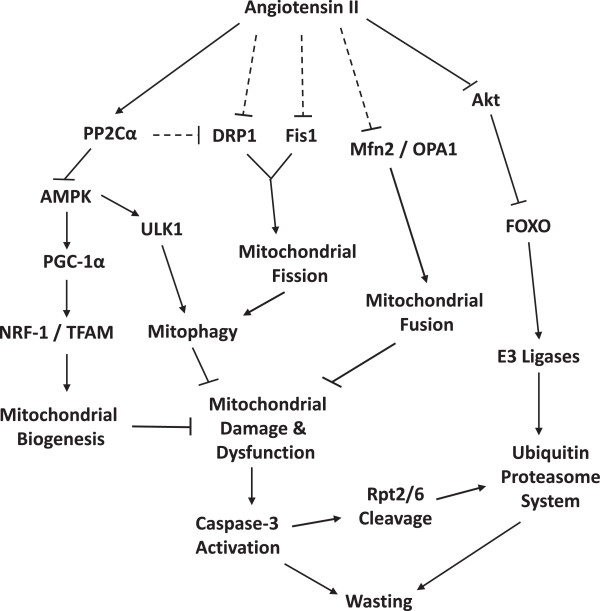
**Proposed model whereby AngII infusion leads to mitochondrial dysfunction and skeletal muscle wasting.** Parallel to the well-characterized AngII-mediated activation of the FOXO-E3-UPS axis via inhibition of Akt, AngII also induces expression of the phosphatase PP2Cα, which dephosphorylates and inactivates AMPK. This leads to reduced PGC-1α, NRF1, and TFAM expression (less mitochondrial biogenesis), and reduced ULK1 activity. The AngII-mediated reduction in ULK1 activation inhibits a critical early step in the autophagy pathway and prevents recycling of damaged mitochondria (mitophagy). AngII also inhibits both mitochondrial fission and fusion through predominately AMPK-independent pathways, which likely contribute to mitochondrial dysfunction caused by elevated AngII. Prolonged mitochondrial dysfunction and energy depletion ultimately leads to release of caspase-3, initiation of apoptosis, and wasting.

Our data show that AngII-mediated skeletal muscle wasting is characterized by impairment of mitochondrial biogenesis, fission, fusion, and autophagy, thereby preventing normal mitochondrial recycling and leading to mitochondrial dysfunction, ATP depletion, and wasting (summarized in Figure 9). Further, we have shown that AngII wasting and its effects on mitochondrial function are mediated at least in part via upregulation of PP2Cα in muscle. Thus, our studies elucidate novel mechanisms underlying AngII wasting and suggest a therapeutic potential for activators of AMPK in wasting conditions where the renin-angiotensin system is activated.

## Abbreviations

AICAR: 5-aminoimidazole-4-carboxamide ribonucleoside; Akt: Protein kinase B; AMPK: AMP-Activated Protein Kinase; AngII: Angiotensin II; ANOVA: analysis of variance; ATP: Adenosine triphosphate; atrogin-1: Atrogin-1/MAFbx, Fbxo32; CHF: Congestive heart failure; complex I: NADH dehydrogenase; complex II: succinate dehydrogenase; complex III: cytochrome bc_1_ complex; complex IV: Cytochrome C oxidase; complex V: ATP synthase; DRP1: Dynamin-1-like protein; Fis1: Mitochondrial fission 1 protein; FOXO: Forkhead box protein class O; Gas: Gastrocnemius muscle; IGF-1: Insulin-like growth factor 1; LAMP1: Lysosomal-associated membrane protein 1; LC3: microtubule-associated protein 1 light chain 3; MARCH5: Membrane-associated RING-CH5; Mff: Mitochondrial fission factor; mtDNA: Mitochondrial deoxyribonucleic acid; mTOR: Mammalian target of rapamycin; MuRF1: Muscle RING-finger protein-1, Trim63; NRF1: nNuclear respiratory factor 1; OCT: Optimal cutting temperature; OPA1: Optic atrophy 1; p62: p62/SQSTM1; PGC-1α: Peroxisome proliferator -activated receptor-γ coactivator 1-α; PP2Cα: Protein phosphatase 2C-alpha; RT-PCR: Reverse transcriptase polymerase chain reaction; SEM: Standard error of the mean; siRNA: Small interfering ribonucleic acid; TFAM: Mitochondrial transcription factor A; UCP3: Uncoupling protein-3; ULK1: UNC-51-like kinase 1; UPP: Ubiquitin proteasome pathway; WB: Western blot.

## Competing interests

The authors declare that they have no competing interests.

## Authors’ contributions

AMT carried out the animal experiments, performed the molecular assays and immunoblots, participated in the design of the study, performed the statistical analysis, and drafted the manuscript. TY performed the quantification of muscle fiber cross sectional area, participated in the design of the study, and helped to revise manuscript. SS performed the immunohistochemical analysis of p62 and LC3 positive punctae in Gas cross sections and participated in the design of the study. PD conceived of the study and participated in its design, coordination, and interpretation of results, and helped to revise the manuscript. All authors read and approved the final manuscript.
